# A Review on Coordination Properties of Thiol-Containing Chelating Agents Towards Mercury, Cadmium, and Lead

**DOI:** 10.3390/molecules24183247

**Published:** 2019-09-06

**Authors:** Geir Bjørklund, Guido Crisponi, Valeria Marina Nurchi, Rosita Cappai, Aleksandra Buha Djordjevic, Jan Aaseth

**Affiliations:** 1 Council for Nutritional and Environmental Medicine, N-8610 Mo i Rana, Norway; 2 Cittadella Universitaria, University of Cagliari, 09042 Cagliari, Italy; 3 Department of Life and Environmental Sciences, University of Cagliari, 09042 Cagliari, Italy (V.M.N.) (R.C.); 4 Department of Toxicology “Akademik Danilo Soldatović”, Faculty of Pharmacy, University of Belgrade, 11000 Belgrade, Serbia; 5 Research Department, Innlandet Hospital, N-2380 Brumunddal, Norway; 6 Inland Norway University of Applied Sciences, N-2411 Elverum, Norway; 7 IM Sechenov First Moscow State Medical University (Sechenov University), 119146 Moscow, Russia

**Keywords:** BAL, DMPS, DMSA, metal chelator, metal ion

## Abstract

The present article reviews the clinical use of thiol-based metal chelators in intoxications and overexposure with mercury (Hg), cadmium (Cd), and lead (Pb). Currently, very few commercially available pharmaceuticals can successfully reduce or prevent the toxicity of these metals. The metal chelator meso-2,3-dimercaptosuccinic acid (DMSA) is considerably less toxic than the classical agent British anti-Lewisite (BAL, 2,3-dimercaptopropanol) and is the recommended agent in poisonings with Pb and organic Hg. Its toxicity is also lower than that of DMPS (dimercaptopropane sulfonate), although DMPS is the recommended agent in acute poisonings with Hg salts. It is suggested that intracellular Cd deposits and cerebral deposits of inorganic Hg, to some extent, can be mobilized by a combination of antidotes, but clinical experience with such combinations are lacking. Alpha-lipoic acid (α-LA) has been suggested for toxic metal detoxification but is not considered a drug of choice in clinical practice. The molecular mechanisms and chemical equilibria of complex formation of the chelators with the metal ions Hg^2+^, Cd^2+^, and Pb^2+^ are reviewed since insight into these reactions can provide a basis for further development of therapeutics.

## 1. Introduction

The US Agency for Toxic Substances and Disease Registry assembles a list of the substances that can cause the most significant problems to human health for their toxicity and potential for human exposure. It should be noticed that this priority list is not a list of “the most toxic” substances, but rather a prioritization of substances based on a combination of their frequency, toxicity, and potential for human exposure. This list is regularly revised to take into account any new information on toxic substances [1].

On these bases, lead (Pb), mercury (Hg), and cadmium (Cd) are classified not only as the most relevant toxic metals, but also as the most relevant toxic substances in general. Furthermore, the World Health Organization (WHO) has also included these three toxic metals in the top 10 chemicals of major public health concern [2].

Therefore, in the present review, we will take into consideration the chelating agents that can be useful for the clinical treatment of Pb, Hg, and Cd intoxication. In particular, since the sulfhydryl (SH) groups of proteins furnish the vehicle for both the toxicity and detoxification of the majority of heavy metal ions, we will take into consideration chelating agents characterized by thiol groups. The review aims to delineate principles that can be used in the search for improved antidotal treatments of these three toxic metals. We will start by recalling the hard–soft properties of these metal ions [3] reported in Table 1.

It can be observed that Cd^2+^ and Hg^2+^, both belonging to group 12 in the periodic table of elements, are classified as soft metal ions, preferring the coordination by ligands characterized by soft groups such as R_2_S, RSH, and RS [4]. On the other hand, Pb^2+^, which belongs to group 14 in the periodic table, is classified as an intermediate metal ion, indicating that above all it will be coordinated by amino groups, even if the interaction with hard oxygen groups and soft thiol groups is observed in a number of complexes. Furthermore, different structural coordination modes characterize these metal ions, such as linear coordination for Hg with thiol groups, or tetrahedral for Cd, but these considerations will be further developed in the last sections of the present paper.

## 2. Exposure and Effects

Table 2 reports some exposure sources and target organs for Hg, Cd, and Pb, which will be discussed in the following lines. 

### 2.1. Mercury

Environmental Hg exists in three chemical forms, viz. elemental Hg (metallic Hg^0^ liquid), inorganic mercuric salts (e.g., Hg chloride, HgCl_2_), and organic Hg compounds (e.g., methylmercury (MeHg, CH_3_Hg) and ethylmercury (EtHg, C_2_H_5_Hg)) [5,6]. Humans are exposed to low chronic levels of mercurial compounds via various routes: Oral, inhalation, and dermal [7], to MeHg mainly through fish, Hg vapor from dental amalgams, and EtHg through vaccines [8]. 

Although organic Hg is regarded as the most frequent and toxic one, elemental Hg is more volatile and, hence, more dangerous than generally perceived. Elemental Hg^0^ exists as liquid metal and can vaporize at room temperature due to high vapor pressure. For example, a worker who stays for about eight hours in a Hg-saturated place can inhale up to about 100 mg of Hg per day [9]. Major sources of elemental Hg emissions to the air are coal burning, metal smelting, crematoriums, waste incineration, and small-scale gold extraction [10]. Emitted Hg vapor is oxidized to ionic form (Hg^2+^) in the air layers, which falls to the ground with rain, often far from the emission point. This makes Hg exposure a global concern. In the soil layers and sediments, Hg has a very long half-life [11,12]. Also, Hg occurs naturally as a result of volcanic activities, forest fire, water movement, etc. [13]. Other important sources of Hg exposure is the use of Hg in measuring instruments and as a disinfectant. Regulatory measures during the last decades have reduced the Hg emissions to the environment significantly [12]. However, still, some hot spots of Hg pollution exist. Mainly in developing countries, Hg poses a threat to the environment and health of nearby living residents. Hence, environmental and human Hg exposure assessments are needed in these regions [11].

The main sources of elemental Hg in humans are Hg released from dental amalgams batteries, and incineration of medical waste [14,15]. In the 1830s, dental amalgam was introduced in the Western World and has since then been subject to recurrent concerns and controversies [16]. Today, many countries, including the Scandinavian countries and Italy, have in principle ceased the use of dental amalgam. However, this filling material is still in widespread use, particularly in developing countries [14]. 

Elemental Hg is oxidized to divalent inorganic Hg in red blood cells and tissues [17]. However, some Hg vapor passes the blood–brain barrier and enters the brain. Elemental Hg, which is highly diffusible and lipid-soluble, is oxidized and accumulated in the human brain. Its half-life in the brain is several years to several decades [18]. Numerous toxic effects and conditions have been linked to Hg vapor exposure. It has been suggested that inhaled Hg vapor from amalgam fillings is a predisposing factor to Alzheimer’s disease [19]. However, this hypothesis remains to be verified [20]. Research has also shown that Hg vapor passes the placenta and is taken up by the fetus. The inorganic Hg concentrations in the placenta and umbilical cord have been found to correlate with the mother’s number of amalgam fillings [21,22]. Dental personnel who are occupationally exposed to Hg have a higher Hg body burden than unexposed individuals., Recently this was reviewed by Aaseth et al. [23] and Bjørklund et al. [24]. Also, dental personnel more often develop uncharacterized symptoms like fatigue, weakness, and anorexia than unexposed people [23]. A similar trend was shown for neurobehavioral effects, like idiopathic disturbances in cognitive skills, affective reactions, and motor functions [24].

In addition to dental personnel, occupational Hg exposure also occurs in the chloralkali industry (if Hg electrodes are used) and in the manufacture of fluorescent lamps and batteries. Adverse effects in the central nervous system of chloralkali workers may persist for ten years or more after high Hg vapor exposure has ceased. Mathiesen et al. [25] found that a group of 70 previously H-exposed chloralkali workers (time passed after the last exposure was on average 12.7 years) had decreased performance on a number of neuropsychological tests compared to an unexposed control group of 52 workers. Comparable results were shown in another study of high-level Hg vapor-exposed workers [26]. It has been demonstrated that adverse Hg effects in the peripheral nervous system are detectable even decades after cessation of exposure [27]. The major clinical feature of chronic elemental Hg poisoning is a triad of tremors, erethism, and gingivitis [28]. Long-term chronic Hg vapor exposure led to mercurial erethism, characterized by excessive shyness and social phobia [29]. In the 19th century, mercuric nitrate was commonly used in felt hat production. At that time in England and the US, the syndrome of erethism was common among exposed hatters. More on historical perspectives of Hg poisoning is given by Brooks et al. and Buckell et al. [30,31]. Apart from the central nervous system toxicity, elemental Hg can affect the human immune system or cause toxic pulmonary, reproductive, or cardiovascular effects [15].

Inorganic Hg^2+^ is absorbed from the gastrointestinal tract after ingestion and also through the skin [32]. The highest inorganic Hg levels are found in kidneys. In the kidneys, inorganic Hg can give many effects, including proteinuria and polyuria. This can further progress into nephritic syndrome [33]. Chronic inorganic Hg poisoning can also cause acrodynia, which is considered a hypersensitivity reaction, characterized by profuse sweating and erythematous rashes of the palms and soles [32].

Of serious concern is Hg exposure via fish and seafood. Mercury bioaccumulates and biomagnifies in the food chain, after biomethylation to MeHg [11,13]. Usually, the MeHg levels increase with the age of the fish [34]. Methylmercury has caused major environmental disasters [35]; the most serious happened in Minamata Bay, in Japan. In the 1950s, the plastic plant belonging to the Chisso Corporation group emitted wastewater containing Hg into this sea bay [36,37]. Over time, this caused a massive Hg accumulation in the food chain. Minamata disease is a neurological syndrome encompassing symptoms of sensory disturbances, ataxia, dysarthria, constriction of the visual field, auditory disturbances, and tremor. Another poisoning incident happened 20 years later in Iraq when the sensory, motor and visual disturbance were developed after ingestion of bread contaminated with organomercury fungicide [38]. After ingestion and rapid absorption of MeHg in the gastrointestinal tract, it circulates in the blood bound to SH-containing amino acid residues and distributed to the central nervous system and other parts of the organism [39,40]. By the use of molecular mimicry, MeHg, bound to the SH group of cysteine, crosses the blood–brain barrier and arrives at glial cells and neurons, where it is slowly converted to inorganic Hg [41]. Epidemiological studies have shown that pregnant women who are exposed to large MeHg concentrations give birth to children with severe brain damage even without having any poisoning symptoms themselves [11,42]. Furthermore, MeHg has been implicated in many neurodegenerative diseases, and a possible role in autism spectrum disorder has been suggested [20,41,43].

According to the International Agency for Research on Cancer, MeHg compounds are possibly carcinogenic to humans (group 2B), while metallic Hg and inorganic Hg compounds are not considered carcinogenic to humans [44].

Mercury compounds exert toxic actions through various mechanisms. Research indicates that toxic effects of organic Hg in the nervous system may be caused or worsened by the oxidized form, Hg^2+^, that binds to the thiol (-SH) groups and thereby alters protein structure and/or inhibits enzymatic functions [41]. Numerous studies have also suggested other mechanisms of Hg toxicity such as induction of oxidative stress, damage of Ca homeostasis, and changes in glutamate homeostasis [6].

### 2.2. Cadmium 

Metallic Cd is, to a significant extent, a by-product of zinc (Zn) production and to some degree, also a by-product from copper (Cu) and Pb production [45]. Since 1990, the annual use of Cd is about 20,000 tons worldwide. Recycling accounts for ca. 18% of the production. A majority of Cd is used in nickel-Cd batteries. Also, Cd is used for corrosion protection of steel (cadmium plating), as a solder and weld metal in alloys, in polyvinyl chloride plastics, and as a pigment in paint colors, different types of paint, and glazes [46]. 

Numerous studies have reported health effects of Cd exposure in the general population, even in subjects without particular industrial exposure. The estimated Cd exposure in many areas, particularly industrial ones, is high enough to represent a human health threat [47,48,49]. Environmental Cd contamination is mainly a result of anthropogenic activities, but can also be of natural origin [50]. Due to high rate soil-to-plant transfer, Cd enters and accumulates in the food chain [51]. In most parts of the world, food is the primary Cd source for non-smokers [47].

Foods rich in Cd include offal, seafood, cocoa powder, and wild mushrooms. However, due to the larger consumption, 80% of Cd in food comes from staples (rice, potato, and wheat) [52,53]. The average daily Cd intake from food is 8–25 μg [50,52]. Currently, the Food and Agriculture Organization (FAO) and World Health Organization (WHO) Joint Expert Committee on Food Additives and Contaminants consider 25 µg Cd per kg body weight/month as a tolerable intake level [54]. However, certain subpopulations can have a much higher Cd intake than the average population (vegetarians, populations that consume rice as a dominant energy source) [52]. Tobacco leaves accumulate Cd. Therefore, cigarette smoke is a significant Cd source in the general population [55]. Cadmium in drinking water typically only contributes a few percent of the total Cd intake [53]. In the air, Cd is present in trace amounts [56]. Therefore, exposure from air generally provides less than a few percent of the total Cd body burden. However, Cd-polluted water and air and even house dust may occur in areas close to some metal industries. Itai-itai disease is the documented case of a mass Cd poisoning in Toyama Prefecture, Japan. What became the world’s first large Cd poisoning disaster started around 1912 and caused a crippling and very painful form of osteomalacia including severe kidney damage and multiple bone fractures [57] The disease got its name due to the pain moans.

After Cd uptake in the body, it is transported via the hepatic portal system to the liver, where Cd induces synthesis of metal-binding proteins, metallothioneins (MTs). Inhaled Cd induces MTs in the lungs, where CdMT complexes are formed directly. CdMTs are released from the liver, enterocytes, and lungs into the systematic circulation. Thus, Cd is transported primarily to the kidneys where it accumulates. A recent review by Satarug presents a detailed overview of Cd kinetics [51]. The half-life of renal Cd is 7–16 years [58] or longer [59]. However, the accumulation of Cd in the organism varies with age, gender, smoking status, and certain co-morbidities. 

Long-term Cd exposure affects many organs. The kidneys have been considered the critical organ of Cd toxicity. Even low-level, long-term Cd exposure may induce various kidney dysfunctions [60]. Also, the liver is critical to Cd accumulation. In both sexes, both acute and chronic Cd exposure is linked to various liver-related diseases [60,61]. Recent epidemiological studies confirm the association between Cd exposure and increased risk of osteoporosis-related fractures [62], which originally was observed during the Itai-itai epidemic in Japan. Also, associations between Cd exposure and cardiovascular diseases [63], reproductive disorders in both sexes [64,65,66], thyroid disorders [67], gestational diabetes, and diabetes mellitus type 2 [68,69] have been shown. Also, Cd may produce hormesis phenomena [70]. IARC classifies Cd and Cd compounds as known human carcinogens [44], based on a causal relationship between exposure and lung cancer. New research has also shown positive associations between chronic Cd exposure and kidney and prostate cancers [71]. Studies have implied a possible role of Cd in pancreatic [72,73], bladder [74], prostate [75], and breast cancer [76].

The mechanisms of Cd toxicity are various and include binding to SH groups, oxidative stress induction [77,78], interactions with bioelements [79,80,81,82], mitochondrial toxicity [83], and altered microRNA expression [84].

### 2.3. Lead

For several decades, the use of Pb-containing gasoline was an environmental and human exposure source of organic Pb compounds [85,86]. Since the 1920s, Pb usually added as tetraethyl lead (TEL) to gasoline caused significant exposure via inhalation of car exhaust [87]. Since Pb is toxic, this gasoline was gradually phased out in most countries of the world. In the US, Pb in gasoline was banned from 1996, and in the EU, organolead was entirely phased out in 2000 [87]. The removal of Pb from gasoline is regarded as one of the major public health triumphs of the 20^th^ century. Also, much work has been done to phase out Pb from various other products completely. To completely eliminate Pb from gasoline and water pipes took a long time but effectively reduced Pb pollution in the environment [86,88]. However, due to the persistence of Pb, it is still present in the environment. Although food generally contains low Pb levels, most of the Pb exposure in many countries nowadays occurs through food and drinking water [86,89]. In Europe, the average exposure via diet is about 0.50 µg Pb/kg body weight/day [90]. Cereal products contribute most to dietary Pb exposure, while Pb in dust and soil can be important sources for children. Also, Pb in old paint dust and soil can be a source of increased Pb exposure for small children, due to their tendency for licking, chewing, and swallowing foreign bodies [91,92]. Residual paints that contain significant quantities of Pb is a problem in many countries of the world, especially for children [93].

Lead exposure mainly happens through the gastrointestinal and respiratory tract. Approximately 30%–40% of Pb from the respiratory tract is absorbed into the bloodstream while the gastrointestinal absorption depends mainly on age and nutritional status [86,94]. Hence, while adults absorb around 10%–15% of ingested Pb, this amount increases up to 50% in infants, young children, and pregnant woman [85,94,95]. Once absorbed, Pb is transported by the bloodstream mainly bound to erythrocytes and distributed to other tissues such as liver, kidneys, brain, lungs, spleen, teeth, and bones. More than 95% of Pb is deposited in skeletal bones while in children, this percentage is less resulting in more Pb in soft tissues [85,86]. Furthermore, Pb passes the placental barrier during pregnancy and can cause damage to the fetus. Concentrations of Pb found in the umbilical cord blood are 80%–100% of the maternal blood levels [96].

Toxic effects of Pb have been detected in virtually every body system. Children are generally more vulnerable to Pb toxicity than adults, especially for neurological Pb toxicity. The most deleterious effects of Pb are detected on erythropoiesis, kidney function, and the central nervous system [85,86,97]. Other toxic effects of Pb include hypertension and hearing impairment, infertility, abdominal pain (“lead colic”), and anorexia [97]. Recent research has linked the level of Pb in drinking water to increased risk of cardiovascular pathologies [98]. For children, Pb exposure may impair cognitive abilities, attention, mental development, and skeletal growth [99]. Also, disturbed blood formation and renal effects may occur in children at relatively low Pb exposure [100]. A lower threshold for children that provides complete safety against Pb poisoning has not been established. The International Agency for Cancer Research classified inorganic Pb as probably carcinogenic to humans (Group 2A) [101].

Many in vivo and in vitro studies have been performed to identify the exact mechanisms of Pb toxicity. Some of them are oxidative stress induction [77,102], binding to sulfur ligands that can affect many enzymes and proteins [77], interaction with bioelements [102], changes in DNA structure, and inhibition of DNA repair [103].

## 3. Endogenous Protective SH-Compounds: Metallothioneins and Glutathione

### 3.1. Metallothioneins (MTs)

The metallothionein (MT) family is cysteine-rich and consists of proteins with low molecular weight (mol. wt. ranging from about 1000 to 14,000 kDa). They are localized intracellularly and can bind both essential and non-essential heavy metals, e.g., Zn, Cu, Cd, Hg, silver (Ag), and As, through the thiol group of its cysteine residues. Approximately 30% of the amino acids in MTs are cysteine. MTs are found in yeasts, plants, invertebrates, as well as vertebrates including humans.

Margoshes and Vallee [104] discovered MT when they purified a Cd-binding protein from the renal cortex of horses. Still, the MTs functions are not entirely understood, but apparently, they protect against the effects of toxic metals as well as being involved in the physiological regulation of Cu and Zn. MTs also protect against oxidative stress [105]. In principle, four isoforms of MTs are present in humans (i.e., MT1 with subtypes, MT2, MT3, and MT4) [106]. All isoforms contain polynuclear metal-sulfur coordination sites. In mammals, MT1 and MT2 are the most common isoforms. In the liver and also in the gut, these MTs get induced by many different metals, especially by Zn ions. MT3 has been found in the central nervous system, and MT4 has been found in epithelial cells. MT3 appears to have a protective function against oxidative stress in the brain [107].

For the production of MT1 and MT2 in the liver, dietary Cu and Zn, as well as the amino acids cysteine and histidine, are needed. The content of the metals in MTs depends on their stability constants and their amount in the body. MTs can tie up many different metals, including Cd, Zn, Hg, and Cu.

Through their binding and release of Zn, MTs can regulate the cellular levels of Zn. According to their affinity constants with MTs, many toxic metal ions, including Hg^2+^, Cu^+^, Ag^+^, Cd^2+^, Bi^2+^, and Pb^2+^, might displace Zn^2+^ from MT. The free Zn, in turn, plays a key role for the binding and activation of transcription factors, especially the metal-regulatory transcription factor 1 (MTF-1), and Zn release induces more MT being synthesized.

Residues of cysteine from MTs may also capture oxidant radicals, including the harmful hydroxyl and superoxide radicals [108]. In such reactions, cysteine oxidizes to cystine, and the Zn ions that were cysteine bound are disconnected. Released Zn can activate the synthesis of further MTs. Various factors induce the gene expression of MTs, including metal exposure, oxidative stress, and glucocorticoids. The MT gene controls the response level of these inducers. MTs can also carry Zn ions between different parts of the cell. When Zn enters into a cell, thionein can take up the trace element and carry it to other cellular parts. This kind of signaling system is considered of particular importance in the brain, where Zn signaling appears crucial both within and between nerve cells [109].

### 3.2. Glutathione (GSH)

Glutathione (GSH), the tripeptide γ-glutamyl-cysteinyl-glycine, is a crucial antioxidant for animals, plants, as well as for some bacteria, in preventing damage on essential cellular components due to some metal ions and peroxides. GSH is the crucial intracellular reducing agent in animal cells. GSH is biosynthesized in the body from its amino acid constituents. Its cysteine thiol group (SH) functions as an electron donor in its interactions with metal ions or oxygen radicals. Cysteine is considered the rate-limiting factor in cellular GSH biosynthesis due to its relatively little presence in foods. The cellular biosynthesis of GSH involves two ATP-dependent steps: First, γ-glutamyl-cysteine is synthesized from L-glutamate and cysteine through the action of the enzyme gamma-glutamyl-cysteine synthetase. This initial step in the GSH synthesis is rate-limiting in the synthesis. In the second reaction, the enzyme glutathione synthetase adds glycine to the γ-glutamylcysteine. GSH exists in two different states, i.e., both as oxidized (GSSG) and reduced (GSH) states. In its reduced state, a reducing equivalent (H^+^+ e^−^) can be donated by the SH group to unstable molecules such as reactive oxygen species. When an electron gets donated, a GSH molecule becomes reactive but reacts readily with another reactive molecule of GSH to create glutathione disulfide (GSSG). Oxidized GSSG is reduced rapidly back to GSH by glutathione reductase, in a reaction where NADPH is used as a donor of electrons [110].

Some important functions of GSH are as follows:

1. It is the principal endogenous antioxidant that the cells produce, it participates directly in the neutralization of ROS and free radicals, and it is a cofactor of the selenoenzyme glutathione peroxidase (GPx).
2. GSH is an important substrate for conjugation reactions, catalyzed by the glutathione-S-transferase enzyme. Thus, in the case of the reactive metabolite formed by a paracetamol overdose, GSH acts as an antidote. GSH can also conjugate and detoxify organometallic compounds, such as MeHg [111].
3. It has important roles in binding, transport, and storing of several metals, thus affecting the homeostasis of metals in biological systems [112].

Here, we will concentrate on its ability to bind toxic metals [113]. Among metals reported to bind to GSH are Cu, Hg, Cd, and Pb. Metals bound by GSH can be exchanged with other ligands. This leads to a fast metal redistribution in the body. The bile appears to be a main excretory pathway for some metal–GSH complexes, which was early indicated for the CH_3_Hg–GSH conjugate. When GSH reacts with a metal, there are two possible outcomes: The metal gets either stabilized as a nonreactive conjugate or the metal, such as transition metals, can undergo a redox reaction paralleled by oxidation of GSH and formation of ROS. Most frequently, GSH binds to metals and protects against the toxicity, e.g., of Hg^2+^.

In healthy tissue and cells, more than 90% of GSH exists in the reduced form (GSH), and less than 10% exists in the disulfide form (GSSG). Increased GSSG/GSH ratio is considered indicative of oxidative stress.

## 4. SH-Containing Chelating Agents: Clinical Use and Environmental Remediation

In 1920, Morgan and Drew suggested the term *chelate* [114], which originates from the Greek word *chele* (claw of a lobster). The term was suggested to be used on the caliper-like groups that function together as two units, which connect a central atom and create heterocyclic rings. In therapeutic use, chelators remove metals from chemical compounds through the formation of complexes. An excellent chelator should be characterized by high solubility in both lipids and water, resistance to biotransformation, ability to reach the sites of metal storage, retain chelating ability at the pH of body fluids, as well as being able to form metal complexes with lower toxicity than the free metal ions [115,116,117]. Unfortunately, even today, most chelators are not able to cross the blood–brain barrier and therefore have limited ability to remove the metals from the brain tissue [4,118].

For more than a century, chelating agents were used by Ehrlich and Werner to decrease the toxicity of arsenic (As)-containing syphilis drugs. During 1920–1940, similar trials to reduce the toxicity of antimony drugs for schistosomiasis and trypanosomiasis were done by Voegtlin et al. [119]. In 1941, Kety and Letonoff used citrate as an antidote towards acute Pb intoxication [120]. This experiment started a new era in treating metal intoxications caused by environmental exposure or genetic disturbances in metal metabolism.

During World War II, Sir Rudolph Peters and colleagues developed the antidote BAL (British anti Lewisite) against the war gas dichlorovinyl arsine (Lewisite) [121]. The next chelator, EDTA (ethylenediamine tetraacetate) was developed for radionuclide decorporation and clinical treatment of Pb intoxication [122]. EDTA must be administered parenterally. Since the intestinal uptake it low, its action is almost exclusively extracellular. To some essential metals, its stability constants are high.

During the 1950s, DMSA (*meso*-dimercaptosuccinic acid) and DMPS (2,3-dimercapto-1-propanesulfonic acid) were used in China [123] and the former Soviet Union [124]. These drugs have been available in the Western world for decades. DMSA is a registered drug in USA and DMPS in Germany. Several decades passed until Western clinicians fully realized their value. Today, they are first-line antidotes in acute or chronic intoxications with many divalent metal salts. The clinical use of DMSA and DMPS in metal intoxications was reviewed by Aaseth [115] and Aposhian et al. [125].

Originally, BAL was a general antidote particularly used in acute As [126] and inorganic Hg poisonings [127]. Earlier, alternative antidotes did not exist. However, BAL is not considered a good chelator today due to its high toxicity. BAL can increase the deposition of Hg and As in the brain [128]. DMSA and DMPS, which are less toxic, are suited for both oral and parenteral administration. Previously, EDTA was used in childhood and occupational Pb intoxications. However, it is no longer recommended due to a possible redistribution of Pb to the brain [129].

### 4.1. BAL (2,3 dimercaptopropan-1-ol)

BAL, which is a dithiol compound, was originally used to treat poisonings caused by the war gas Lewisite [121]. It competes successfully with protein SH groups for the treatment of Lewisite and other As poisonings. Also, BAL forms stable chelates with other toxic metals. For several decades after World War II, it was recommended for inorganic Hg, arsenic, antimony, gold, and bismuth poisonings [130]. In cases of elevated intracranial pressure and encephalopathy due to acute Pb poisonings, BAL was earlier recommended given i.m. in the initial phase in combination with calcium EDTA infusion [131]. However, this advice is now outdated [129]. BAL has a short half-life. Within four hours, its metabolism and excretion are completed. Given in full dose, BAL has severe and sometimes very serious adverse effects, including elevated blood pressure followed by tachycardia. Due to the high toxicity, BAL is currently only used for a few days in life-threatening and acute Pb or As intoxications [132]. Due to BAL’s small safety margin, tendency to redistribute toxic elements to the brain, and painful intramuscular injections, it is, in most cases of metal poisoning, replaced by DMSA and DMPS [4]. In the few cases when BAL is used, it is given as deep intramuscularly injections (2.5 mg BAL/kg every four hours). BAL is contraindicated in the treatment of poisonings with Cd, as well as alkyl- and aryl-Hg compounds.

### 4.2. DMSA (meso-dimercaptosuccinic acid, Succimer)

DMSA (*meso*-dimercaptosuccinic acid, Succimer) and DMPS (2,3-dimercaptopropane-1-sulfonic acid, Unitiol) are water-soluble dithiols, derived from dimercaprol (2,3-dimercapto-1-propanol, BAL) [133].

DMSA can be administered as intravenous, oral, transdermal, or suppository preparations. Plasma and whole-blood half-lives and urinary elimination half-life of DMSA are less than four hours in humans [134,135]; longer in Hg-intoxicated persons [136]. Since DMSA is hydrophilic, it can be administered orally. About 20% of it is absorbed in the gastrointestinal tract, depending on the gut’s health status. About 95% of the absorbed drug bind to plasma proteins (albumin). It probably binds by one of its SH groups on a cysteine residue of albumin. Thereby, DMSA leaves its other SH group free to bind metals [137]. Of the free drug, only a tiny amount remains [135]. Only 10%–25% of the oral application is excreted through urine. The other part is excreted via feces. In the body, it is largely metabolized to various disulfides with cysteine [138,139,140]. DMSA is confined to the extracellular space and does not enter red blood cells [134]. It increases the excretion of Ag, Cd, Pb, and Hg via the urine. Also, it can remove MeHg and Pb from animal brains [141]. Children have a lower renal clearance for DMSA than healthy adults [135].

DMSA is considered the drug of choice in organic Hg poisonings [130,142]. It does not pass the blood–brain barrier but appears to indirectly reduce the MeHg brain burden by changing the brain-to-blood equilibrium [129,141,143,144,145].

Compared to other dithiol antidotes, DMSA is less toxic [146]. It also has the advantage that practically no essential metal is lost (Fe, Ca, magnesium (Mg)). Only minor changes in Cu metabolism is observed [144]. Side effects range from skin reactions, mild neutropenia, and gastrointestinal discomfort, to increased liver enzymes [138]. Rare adverse effects of DMSA treatment are toxic epidermal necrosis and mucocutaneous eruptions [143,147].

In 1991, Roels et al. [148] found that intake of two grams DMSA significantly elevated urinary Hg excretion in occupationally Hg-exposed people. A meta-analysis by Miller et al. [137] proved DMSA safe and efficient. They concluded that DMSA, due to its efficacy, urinary Hg excretion, and safety, is the preferred antidote against Pb. On average, oral DMSA treatment increases the excretion of Pb by a factor of 12. In 17 Pb-poisoned adults, DMSA reversed the gastrointestinal and neurological symptoms of Pb poisoning [140]. Excretion of Pb after DMSA administration increased significantly in chronically exposed adults and children [130]. A patient reported by Gustavsson and Gerhardsson had severe symptoms of Pb poisoning from an accidentally ingested Pb bullet during a game meal. Years after the incident, the patient was cured after removal of the bullet from the bowel and over one year of therapy with DMSA [149].

### 4.3. DMPS (2,3-dimercaptopropane-1-sulfonic acid, Unitiol)

In different countries, DMPS can be prescribed as a drug in capsules for oral antidote treatment (one capsule Dimaval^®^ contains 100 mg DMPS) or in ampoules for intravenous treatment (5 mL ampoule DMPS-Heyl^®^ contains 250 mg DMPS). In Germany, DMPS is a registered drug for treatments of Hg intoxication. However, it is not an approved drug in the US, so unless the U.S. Food and Drug Administration gives special permission, DMPS cannot be legally used by physicians in the US, nor can pharmacies compound it [150]. The daily dose is usually 3–10 mg DMPS/kg body weight.

Many studies have proven its efficiency to chelate toxic metals in the body [145]. DMPS is considered an optimal antidote in inorganic Hg poisonings [130]. For Pb and organic Hg poisonings, it is less efficient than DMSA [151].

The fraction of absorption of oral DMPS is less than 40% [152]. DMPS can be administered orally or intravenously. DMPS converts quickly to disulfide form. The half-life in different organs for DMPS is approximately 20 min [153]. In animal experiments, relatively small DMPS concentrations were detected in the brain and other organs [144]. DMPS is primarily excreted via urine and to some part via the bile. Its use is usually accompanied by some loss of Zn and Cu. Therefore, it is recommended to monitor and replace these trace elements before and after the treatment [154]. DMPS, which is hydrophilic, is distributed primarily in the extracellular space, but a fraction can pass into the intracellular compartment [130]. DMPS removes Hg better from the kidney than DMSA. In cases of acute poisonings with inorganic mercuric salts, DMSA is considered the drug of choice [141,144].

### 4.4. Penicillamine (D-2-amino-3-mercapto-3-methylbutanoic acid)

d-Penicillamine (d-2-amino-3-mercapto-3-methylbutanoic acid, Cuprimine) was introduced in the racemic form (PA) for the treatment of Wilson disease by John Walshe [155]. It is a product of penicillin degradation. Its structure represents a dimethylated cysteine where two methyl groups surround the SH group and give the molecule a higher resistance than cysteine against in vivo interactions. The d-form of penicillamine has fewer side effects than the l-form and is currently the preferred therapeutic form [156]. Penicillamine’s distribution volume consists primarily of the extracellular space. Accidentally, penicillamine may give rise to serious adverse effects [157]. Also, d-penicillamine has been used as an antidote in Hg and Pb poisonings, before DMSA and DMPS were clinically introduced [130].

### 4.5. Lipoic and dihydrolipoic acids

Alpha-lipoic acid ((R)-5-(1,2-Dithiolan-3-yl)pentanoic acid, LA) is an organo-sulfur compound also known as thioctic acid. It is usually produced in the body, and it is essential for aerobic metabolism. The reduced form of LA, called dihydrolipoic acid (DHLA), contains a pair of thiol groups. Here again, the R-enantiomer is the biologically and therapeutically active form. DHLA has high affinity to Hg and has been proposed as an effective Hg antidote [158,159].

### 4.6. MiADMSA (monoisoamyl 2, 3-dimercaptosuccinic acid)

Monoisoamyl 2, 3-dimercaptosuccinic acid (MiADMSA) is currently in development as a future chelating agent. In contrast to DMSA and DMPS, which effectively remove extracellularly distributed Cd [160], MiADMSA can also chelate intracellular Cd [161]. This analog of DMSA can cross biomembranes and is more efficient than DMSA in reducing the burden of subchronic and acute arsenic intoxications [162]. Also, MiADMSA has lower toxicity than DMSA [163]. When used together with N-acetylcysteine, it reduces significantly oxidative stress during chelation therapy [164].

### 4.7. Thiocarbamates (Diethyldithiocarbamate and Derivatives)

Depending on the lipophilicity of a metal-chelator complex, chelating agents may change the metal’s organ distribution, and thereby potentially increase its toxicity. Diethyldithiocarbamate (DDC) was originally suggested as an efficient chelator for acute Cd intoxication, as parenteral DDC administration decreased mortality induced by parenteral Cd in animal experiments, even at protracted time after Cd administration [165]. In general, DDC forms highly lipophilic complexes with divalent metal ions. Increased brain deposition caused by exposure to DDC has been documented for organic and inorganic Hg [166], as well as for Pb [167].

However, some derivatives of DDC with higher molecular weight appear promising in mobilizing aged Cd deposits. Thus, N(methoxybenzyl)-Dglucamine dithiocarbamate in studies on animals effectively reduced the retention of Cd both in organs and the entire body. This agent’s effects were shown to be less pronounced in younger than in older animals. The highest administered Cd fraction retained in the liver, and the strongest chelation therapy effect observed was also on liver deposits. Mobilized Cd was almost exclusively excreted through feces [168].

## 5. Combination of Chelating Agents

It has been shown that DMSA, used in combination with Monensin (the sodium salt of monensic acid, an antibiotic used in ruminant animal feeds [45]), is even more efficient than when it is used alone, in particular in removing Pb deposited in the brain. The suggested mechanism to explain this is that there may be a cotransport of Pb and OH ions leaving the cells, in exchange with external sodium ions; this would promote transport of intracellular Pb to extracellular DMSA, thereby enhancing its effectiveness [169]. Thus, Monensin acts as a shuttling agent for DMSA [118]. The combination of EDTA and BAL was, for many years, recommended in inorganic Pb poisonings [170,171,172]. It is reasonable to assume that the tightly bound Hg-ions in the brain after long-term Hg^0^ vapor exposure, to some extent, can be mobilized by using minor doses of BAL as a brain-to-blood shuttle, in combination with DMPS to promote the final elimination from the body [118]. Furthermore, a combination of MiADMSA and DMSA may be proven more efficient than each agent alone to promote Cd mobilization, although this metal is tightly bound to MTs intracellularly [160].

In cases with acute Hg salt poisonings, venous hemodiafiltration (CVVHDF) is suggested in combination with DMPS. In a case reported by Dargan, a man who had severe Hg poisoning after the ingestion of one gram mercuric sulfate in a suicide attempt, presented acute hematemesis and deteriorated rapidly. The treatment with the combined strategy saved him. He developed no neurological symptoms and was symptom-free five months after being 50 days under hospital care [173].

## 6. Chemical Features of BAL, DMSA, DMPS, Penicillamine, Lipoic Acid, Dihydrolipoic Acid, and their Metal Chelates

### 6.1. Protonation of the Thiolate Anions

In the following, we report the acidic properties of the chelating agents BAL, DMSA, DMPS, penicillamine, lipoic acid, and dihydrolipoic acid, together with those of the simpler ligands thioglycolic acid and thiomalic acid related to DMSA, to obtain insight into the behavior of the parent molecules. Table 3 reports selected protonation constants of these ligands (those concerning the mercapto groups are marked in red) together with their structure, the used acronyms, the formula, and the molecular weights. The protonation constants are of particular importance since they determine the biological properties of a drug, such as its solubility, absorption, cell penetration, and bioavailability. Furthermore, protonation constants are also of primary importance in determining the speciation of the complexes formed with the toxic metal ions of interest.

#### 6.1.1. TGA and TMA

**Figure 1:**
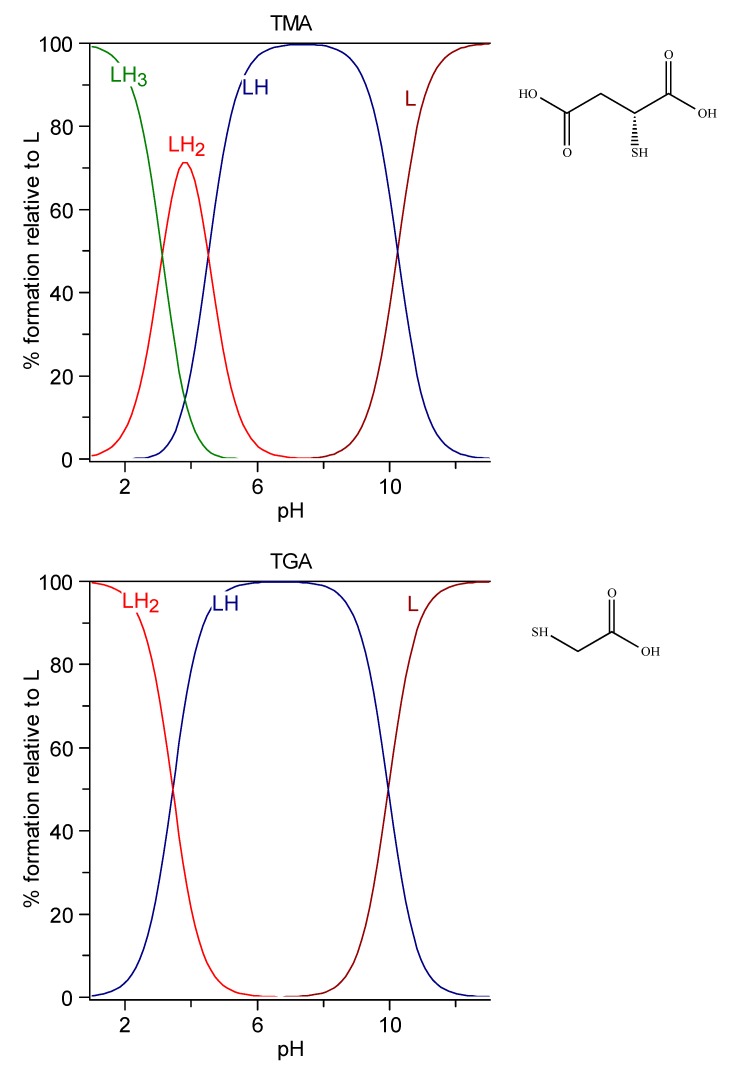
Speciation plots of TGA (top) and TMA (bottom).

The speciation plots of thioglycolic acid (TGA) and thiomalic acid (TMA) are reported in Figure 1. In the case of TGA, the negatively charged [LH]^−^ species with protonated mercapto group is the prevailing one at physiological pH. In the case of TMA, the [LH]^2-^ species, which has lost two protons from the carboxylic groups, is the prevailing one.

#### 6.1.2. BAL

**Figure 2:**
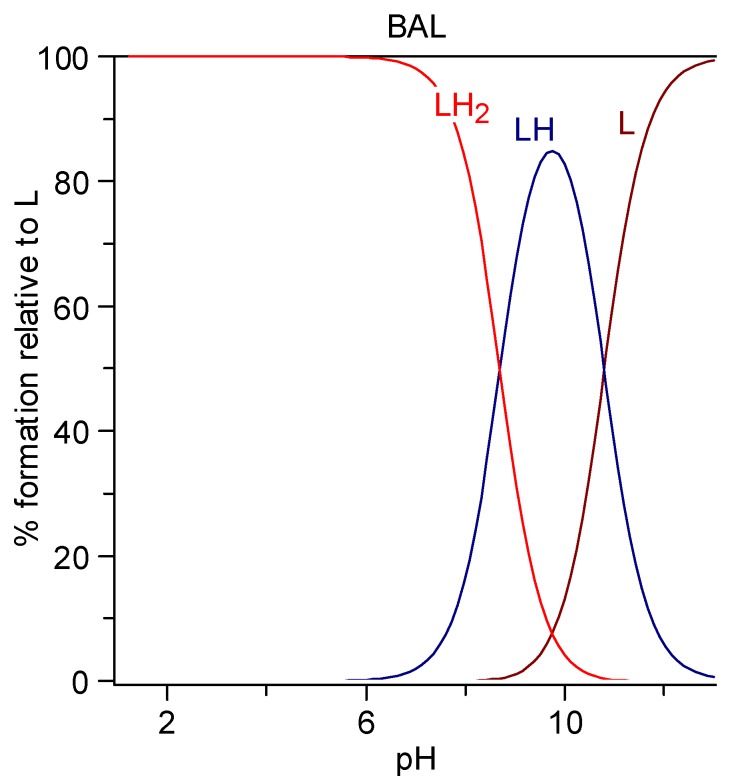
Speciation plots of British anti-Lewisite (BAL).

BAL (2,3 dimercaptopropan-1-ol, dimercaprol) is a viscous oily liquid with a pungent odor of mercaptan, density 1.23985 g/mL, solubility in water 87 g/L, or 0.7 M [179,180]. It is characterized by two protonation constants (log K_1_ 10.8 and log K_2_ 8.7 obtained as the mean values among the cases reported at 25 °C and 0.1 M in the IUPAC Stability Constant Database [181]. The speciation plot is reported in Figure 2. The completely protonated form LH_2_ is prevalent at pH 7.4 (95.2%) together with the monoprotonated form (LH)^−^ (4.8%).

#### 6.1.3. DMSA

**Figure 3:**
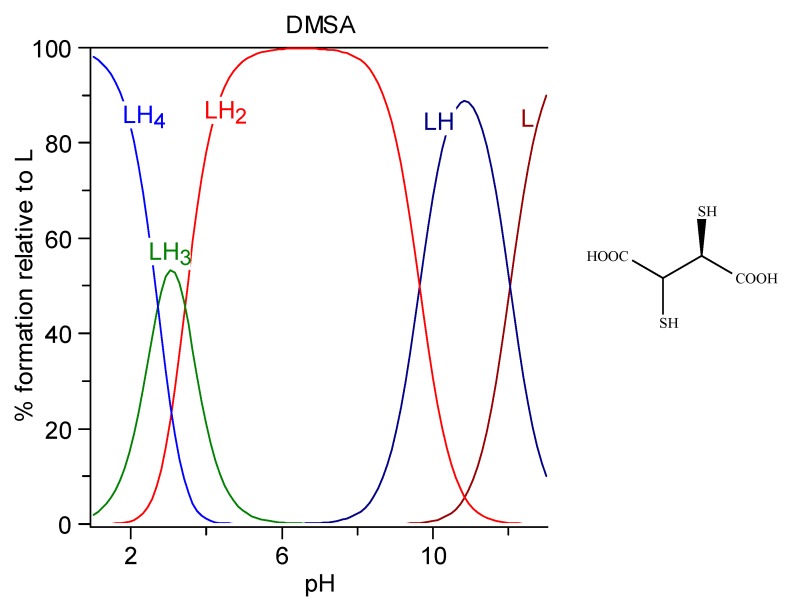
Speciation plots of DMSA.

DMSA (*meso*-2,3-dimercaptosuccinic acid, Succimer) is a white crystalline powder with mercaptan odor and taste, water-solubility 2.43 g/L (DMSA is sparingly soluble; it must be titrated with alkali to pH 5.5 to go into solution, i.e., it must be salified on both carboxylic groups, as can be seen in the speciation plot in Figure 3), log P = −0.3. It is characterized by four protonation constants, logK_1_ = 12.05, logK_2_ = 9.65, logK_3_ = 3.43, and logK_4_ = 2.71. Its formula and the related speciation plot are presented in Figure 3. The form [LH_2_]^2−^ that has lost both the carboxylic protons is the prevalent form at pH 7.4 (99.4%).

**Figure 4:**
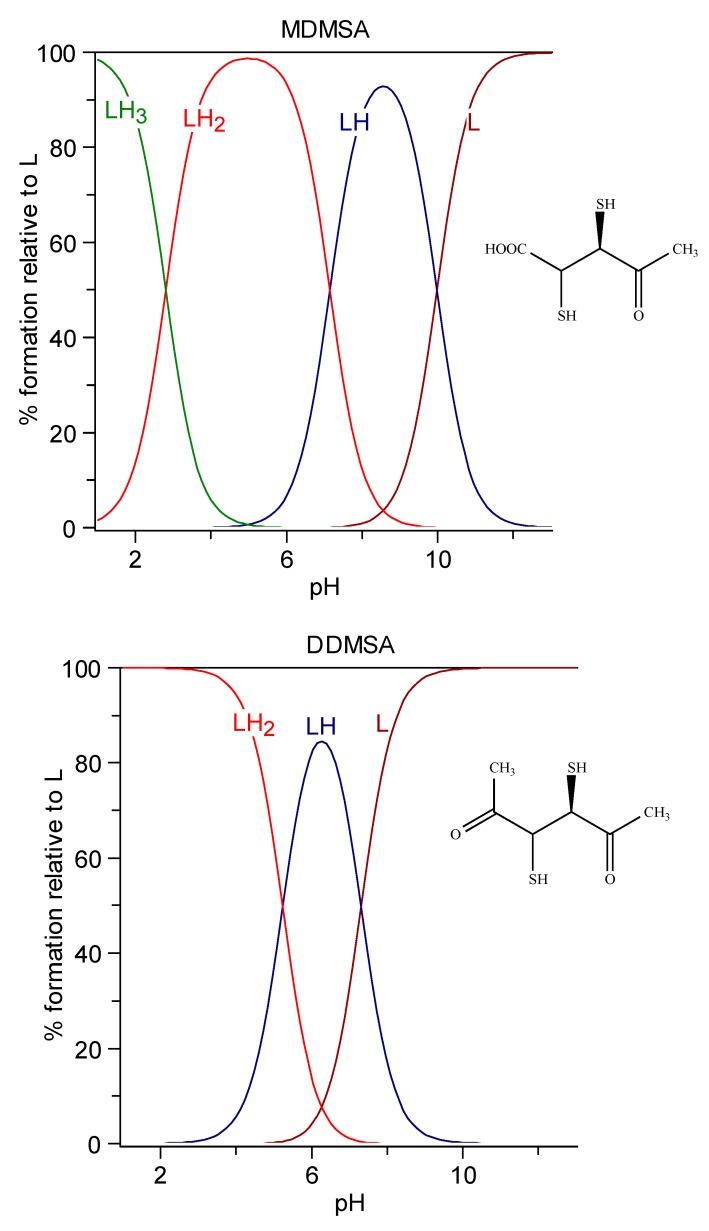
Speciation plots of methyl-DMSA (top) and dimethyl-DMSA (bottom).

Esters of DMSA, more effective than DMSA at clearing Hg and Cd from the intracellular space, have been developed successively. Their better chelating properties are attributed to their higher lipophilicity, favoring cell penetration. Despite the esterification of carboxylic groups, the net charge at pH 7.4 is almost the same as that of the parent molecule, due to the resulting increased acidity of mercapto groups (Figure 4) [174].

#### 6.1.4. DMPS

**Figure 5:**
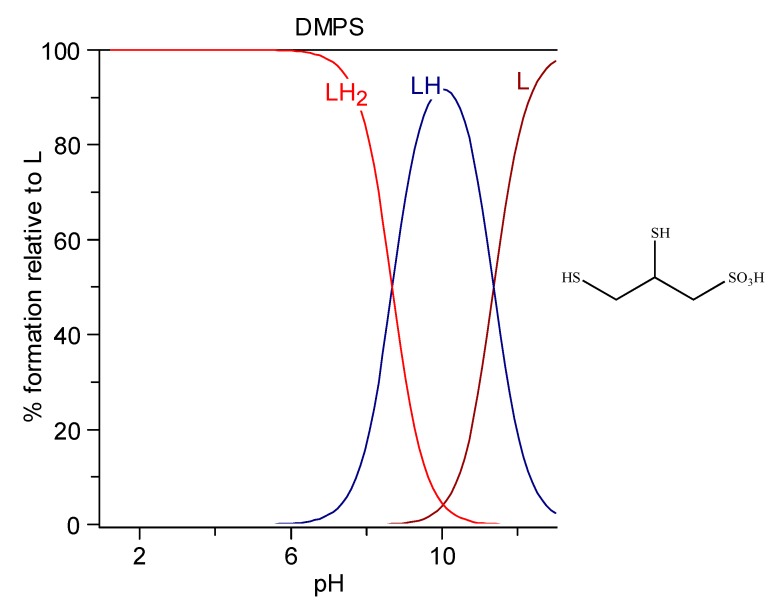
Speciation plots of DMPS.

DMPS (2,3-dimercaptopropane-1-sulfonic acid) is used as the sodium salt that presents as a white crystalline powder with one molecule of crystallization water of general formula C_3_H_7_NaO_3_S_3_ · H_2_O, MW 228.26, produced in Germany by Heyl Chemisch-pharmazeutische Fabrik GmbH, with the trade name of Dimaval^®^. It has a high water solubility of 350 g/L, corresponding to a 1.54 M solution. It is commercially available as ampules for injection (250 mg as C_3_H_7_NaO_3_S_3_ in a sterile solution under nitrogen atmosphere to protect against oxidation) or as 100 mg capsules for oral use (always as C_3_H_7_NaO_3_S_3_). It is characterized by two protonation constants, logK_1_ = 11.62 and logK_2_ = 8.53, behaving in the sulfonic group as a strong acid. Its formula and the related speciation plot are presented in Figure 5. The form [LH_2_]^−^ deprotonated on the sulfonic group is the prevalent form at pH 7.4 (99.4%) [182].

#### 6.1.5. d-penicillamine

**Figure 6:**
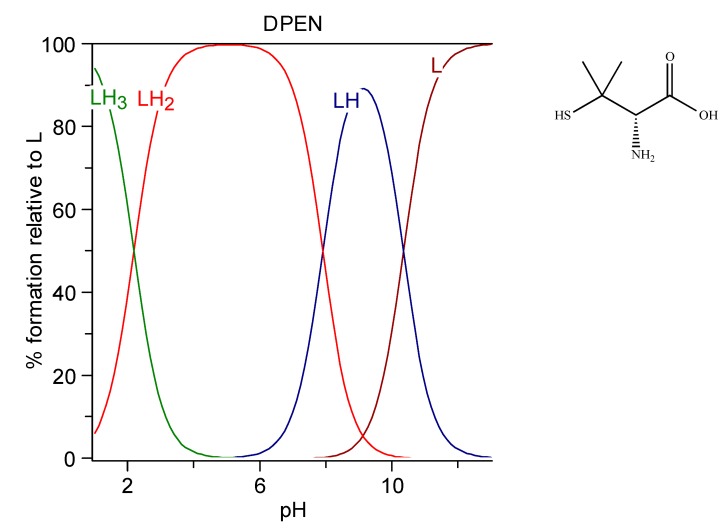
Speciation plots of d-penicillamine (DPEN).

d-penicillamine (DPEN), d-2-amino-3-mercapto-3-methylbutanoic acid, Cuprimine, is a colorless crystalline powder with a weak odor of sulfur-containing amino acids. It is relatively soluble in water [183]. It is characterized by three protonation constants, imputable to SH, NH_3_^+^, and COOH groups respectively, logK_1_ = 10.8, logK_2_ = 8.1, and logK_3_ = 2.2. These were obtained as the mean values among the cases reported at 25 °C and 0.1 M in the IUPAC Stability Constant Database [181]. The speciation plot of DPEN is reported in Figure 6. The zwitterionic form HS-C(CH_3_)_2_-CHNH_3_^+^–COO^−^ (LH_2_ 76.4%) and the negatively charged HS-C(CH_3_)_2_-CHNH_2_–COO^−^ (LH 23.6%) are the species existing at physiological pH.

#### 6.1.6. Lipoic and dihydrolipoic acids

Lipoic acid, (R)-5-(1,2-Dithiolan-3-yl)pentanoic acid, known as α-lipoic acid, (LA) or thioctic acid, appears as yellow needle-like crystals. It is reported to be very slightly soluble in water (0.224 g/L, corresponding to a solution 1.16 mM).

Since no protonation constant in water solution is reported in the literature for the carboxylic acid, we determined it at 25 °C and 0.1 M NaCl ionic strength through potentiometric measurements. The lipoic acid was a reagent-grade Aldrich product, used without further purification. The operating conditions were those generally used by our research group [184]. The solution of ligand to be titrated was obtained by dissolving an excess of lipoic acid in 0.1 M NaCl solution in an ultrasound bath for 3 h. This solution resulted in 6.1 mM of lipoic acid, corresponding to a solubility of 1.26 g/L, about six times greater than that reported above. The log K of protonation resulted in 4.704 (1), very similar to the value 4.73 for DHLA found by Bonomi et al. at the same experimental conditions [178].

**Figure 7:**
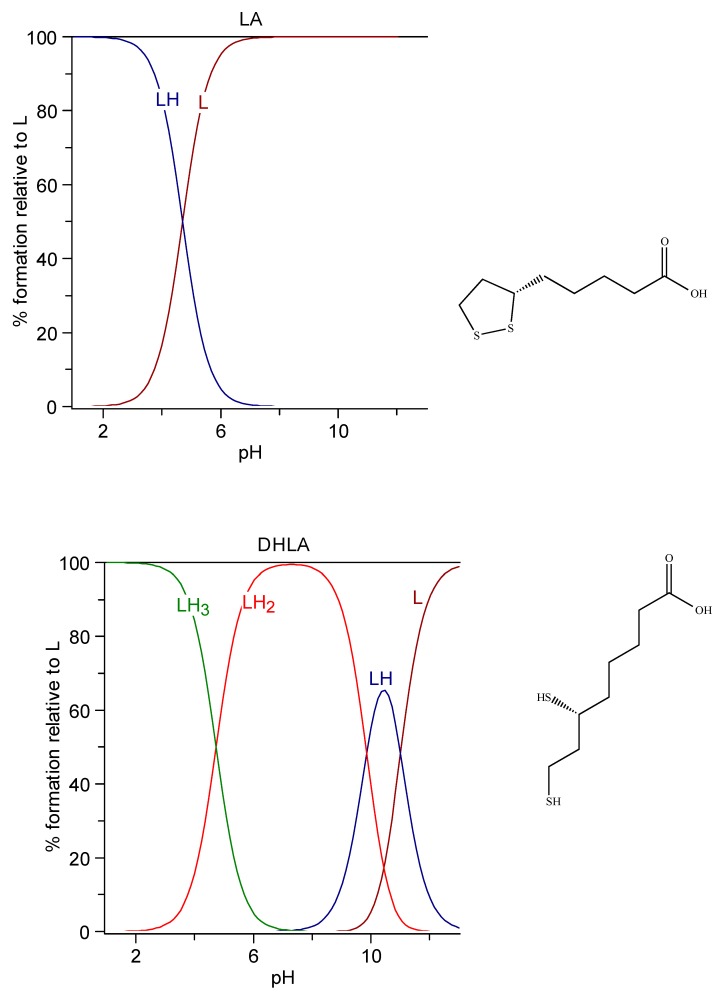
Speciation plots of Lipoic acid (top) and dihydrolipoic acid (DHLA) (bottom).

Dihydrolipoic acid (DHLA), 6,8-Bis(sulfanyl)octanoic acid, is the reduced form of lipoic acid. It is freely soluble in water (103 g/L corresponding to a 0.49 M solution), it has a log P 2.24, and it is characterized by the three protonation constants logK_1_ = 11.02, logK_2_ = 9.86, and logK_3_ = 4.73 at 25 °C and 0.1 M ionic strength [178]. The speciation plots of lipoic acid and DHLA are reported in Figure 7.

Some general features on the results for the protonation constants of mercapto groups (Table 3) can be remarked. In molecules with a single SH group, the log K value presents a low variability, ranging from 9.96 for TGA to 10.35 for DPEN. There is instead a large difference in both the first (10.38 BAL, 11.35 DMPS, and 12.05 DMSA) and also the second protonation constants (8.7 BAL, 8.69 DMPS, and 9.65 DMSA) when two SH groups are present in the molecule. These differences are to a large extent due to the different charges on the entire molecule. The solid-state structures of a number of examined chelating agents are presented in Table S1.

### 6.2. Complex Formation Reactions between Hg^2+^, Cd^2+^, and Pb^2+^ and Thiol Chelating Agents

As remarked in a previous work [185], the literature reports only a few data on the complex formation equilibria between Hg^2+^, Cd^2+^, Pb^2+^, and the thiol chelating agents in Table 4. The literature complex formation constants are reported in Table 4. In the same table, the corresponding pM values are reported for each ligand–metal ion system. Irrespective of the complexation model, the pM values permit us to develop some consideration on the general behavior of each metal ion. The speciation plots for the systems are shown in Figure S1.

In the case of Hg complexes, apart from the pHg value 8.24 for the Hg–TMA complexes studied by Lenz and Martell [189], the pHg values are all extremely high, regardless of the number of SH groups in the molecule. Contrarily, the pM values of Cd^2+^ and Pb^2+.^complexes with the ligands containing two SH binding groups are higher than those with ligands with a SH group alone. The pCd and pPb values are lower than the corresponding pHg values of more than 20 pM units. In a work of Basinger et al. [186], the authors stated “(a) the structural chemistry of Hg^2+^ complexes with thiol-containing ligands, (b) the stability constants for such systems and (c) the relative efficacy of 29 compounds as antidotes for Hg poisoning has been carried out to determine the structural requirements for Hg^2+^ antidotes. This leads to the suggestion that instances in which two thiol groups on the same chelate molecule are simultaneously bonded to the same Hg^2+^ species in a complex, water-soluble or otherwise, are not found, and Hg^2+^ species bearing two sulfur donors generally have a bond angle of 180° or so between these two bonds. It was hypothesized, from this, that chelating agents bearing a single SH group might be almost as effective as Hg antidotes as those bearing two groups. Data on the stability constants and antidotal effectiveness are presented for such structures, but in general, molecules which do not have the potential ability to chelate are also inferior as antidotes. From the data assembled, it would appear that the presence of a second donor group is required. Because of the lability of the Hg-SH bond, this second site seems necessary to provide the required kinetic stability for the complex. In the case of dithiols, the Hg may move back and forth from one sulfur donor site to another but may not (and possibly cannot) bind firmly and simultaneously to both donor sites.”

**Figure 8:**
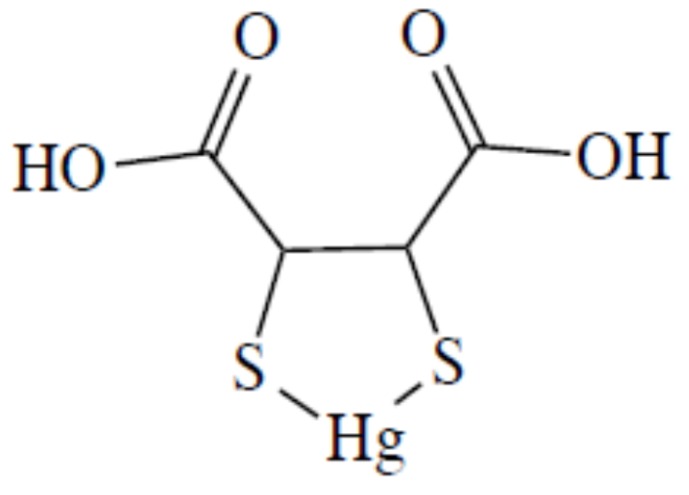
The molecular formula of DMSA–Hg complex proposed by Rivera et al. [193].

In their potentiometric equilibrium study on the complex formation reaction between Hg^2+^ and dithiol chelating agents, Rivera et al. [195] pointed out the formation of a 1:1 complex in which both the two thiol groups were involved in coordination. In the case of DMSA, they proposed the structure in Figure 8, as well as Aposhian et al. [125] six years later.

**Figure 9:**
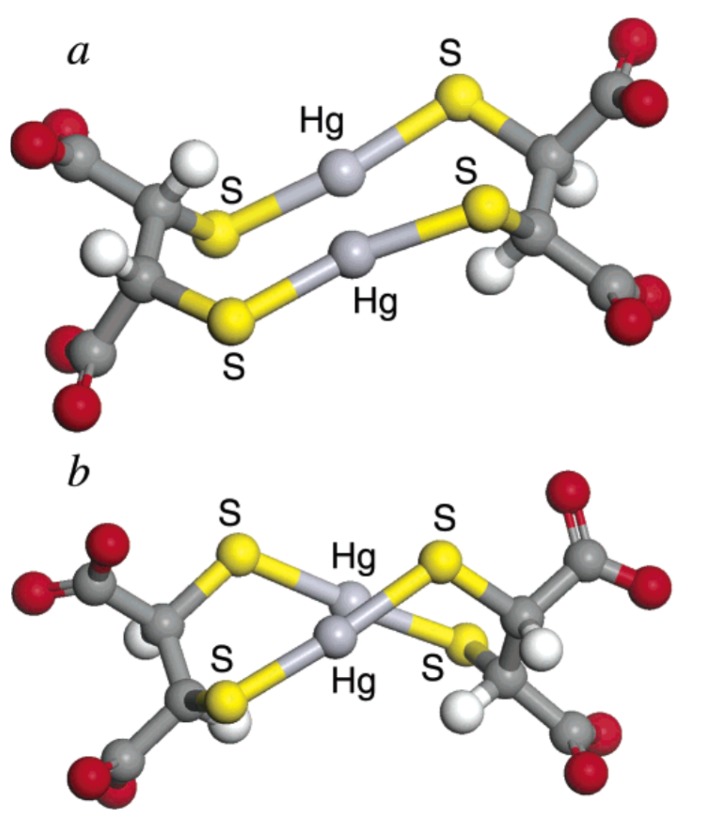
Calculated structures of the two diastereomers of the smallest possible DMSA:Hg^2+^ complex. The carbon atoms are depicted as dark gray, oxygen atoms as red, hydrogen atoms as white, mercury atoms as light gray, and sulfur atoms as yellow. Reproduced from reference [144].

In a work of 2004, George et al. [145] presented a study of the solution chemistry of mercuric ions with DMSA and DMPS, employing X-ray absorption spectroscopy and density functional theory calculations (DFT). The reported complexes were Hg_2_L_2_ and Hg_3_L_3_ for DMSA, and Hg_2_L_2_ for DMPS, at 1:1 M/L ratio. With DMPS at 1:4 M/L ratio, HgL_4_ was also observed. Contrary to established thinking reported above, the authors stated that the two functional groups of the chelator molecule cannot bind a common atom of *Hg* because the distance between the two sulfur atoms in the molecule of DMSA or DMPS does not allow the accommodation of a linear two-coordinate S–Hg–S species (Figure 8). Therefore, they concluded that more complex structures must be occurring with at least two DMSA or DMPS molecules and at least two Hg atoms, and based on DFT studies; they proposed the structures in Figure 9 in which the two coordinated species are both close to linear.

Chekmeneva et al. used differential pulse voltammetry (DPV) and electrospray ionization mass spectrometry (ESI-MS) to the study of the binding of DMSA, DMPS, and DPEN with Hg^2+^ metal ions [196]. The use of voltammetric titrations allowed obtaining a thorough picture of the complexation schemes in a concentration range extremely low. The main formed complexes were Hg(DPEN)_2_, Hg_2_(DMSA)_2,_ and Hg(DMPS)_2_. Further minor species were also evidenced; Hg_2_L species for DMSA and DMPS by DPV; HgL_2_, Hg_2_L_3_, Hg_3_L_3_ for DMSA; and Hg_2_L_2_, Hg_2_L_3_, Hg_3_L_3_ for DMPS by ESI-MS.

The system Hg-DPEN was previously studied by Koszegi-Szalai and Paal [197], who by potentiometric methods and Raman spectroscopy showed that variously protonated Hg(DPEN)_2_ complexes are the dominant species in a wide range of pH, and by Leung et al. [198]. These last authors studied the complexation of Hg^2+^ by DPEN using EXAFS and ^199^Hg NMR, giving evidence of the formation of HgL_2_, and HgL_3_ in excess of DPEN.

**Figure 10:**
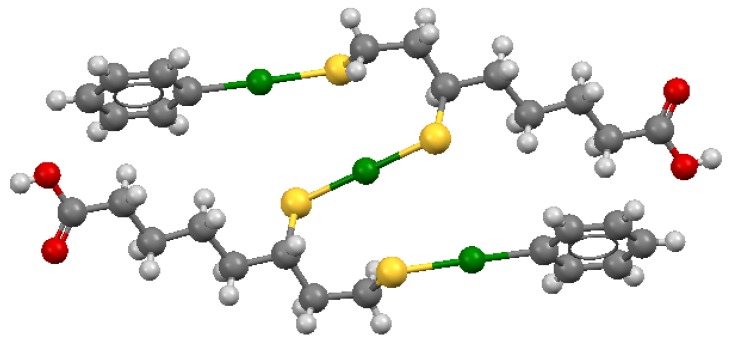
Mercury in green, sulfur in yellow, and oxygen in red. Coordinates obtained from the Cambridge Structural Database (Reference Code LIMNUQ; the image was created with Mercury3.5.

The structure of the complex formed by two DHLA molecules, one Hg^2+^ ion, and two phenyl-Hg groups, shown in Figure 10, presents a similar linear binding coordination mode [199].

The linear coordination of Hg^2+^ is also indirectly reported by Chekmeneva et al. [200], who remarks the formation of 1:1 and 1:2 metal:ligand complexes through DPV, and give evidence of the more complex stoichiometries Hg_2_(DHLA)_2_, Hg(DHLA)_2_, Hg_2_(DHLA)_4_, and Hg_4_(DHLA)_4_ through ESI-MS spectroscopy. The formation of polynuclear species can be explained, taking into consideration the short distance between the two thiol-groups in the chelating agent. Only the participation of several ligand moieties can provide a stable linear conformation with two thiol groups for each Hg^2+^ metal ion. The structures presented in Table S2 for a number of Hg–SH complexes confirm the preferred linear coordination of the Hg^2+^ metal ion.

In the case of Cd^2+^ complexes with thiol-containing ligands (Table 4), a significant difference can be observed between the pCd values of ligands containing a single SH group (TGA 6.00, TMA 7.78, DPEN 8.61, 8.46) and those containing two vicinal SH groups (DMSA 11.48, DMPS 13.24, 12.14). In this last case, the possibility of forming tetrahedral chelates favors their stability. This is confirmed by the solid-state structures presented in Table S3. In a recent study, Jahromi et al. [201] report a structural study of the solution equilibria between Cd^2+^ and DMSA and DMPS. They make use of different techniques, X-ray absorption spectroscopy (XAS), size exclusion chromatography, and density functional theory (DFT). The results indicate complex chemistry consistent with both DMPS and DMSA acting as true chelators, using two thiolates for DMPS and one thiolate and one carboxylate for DMSA [201].

A behavior analogous to that of Cd^2+^ is presented by Pb^2+^ complexes, which show a definite difference among the ligands bearing a single SH group (TGA 6.95, TMA 8.9) and those with two vicinal SH groups (DMSA 11.45 and DMPS 12.00), while DPEN presents an intermediate behavior with pPb 10.5, presumably for the involvement in coordination of NH_2_ or COO^−^ groups. A paper by Gala Morales et al. [202] presented a work where cyclic voltammetry (CV) and DPV were used to study the complex formation of Pb^2+^ with DMSA and DMPS. Multivariate curve resolution was applied to voltammetric results to estimate the stoichiometries and stability constants of the formed complexes. In both systems, the ML_2_ was found as the predominant species. Table S4 presents a number of solid-state structures of Pb^2+^ complexes with mercapto ligands.

## 7. Conclusions

Based on clinical, experimental, and in vitro studies discussed in the present review, some up-to-date recommendations can be given with regard to drugs of choice in metal intoxications with Hg, Cd, and Pb (Table 5). The metal chelator DMSA is considerably less toxic than the classical metal antidote BAL, and today DMSA is the recommended agent in poisonings with Pb and organic Hg. Its toxicity is also lower than that of DMPS, although DMPS is the recommended agent in acute poisonings with mercuric salts. We have suggested that intracellular Cd deposits and cerebral deposits of inorganic Hg, to some extent, can be mobilized by a combination of antidotes (Table 5), but clinical experience with such combinations are lacking. The agent MiADMSA is not yet commercially available, and its possible use in Pb poisonings must await further research. Moreover, the clinical combination of minor doses of BAL with DMPS in cases of Hg vapor overexposure is also insufficiently studied.

## Figures and Tables

**Table 1:** Classification of the toxic metals, and the coordinating groups, according to their hard, intermediate (borderline), and soft character. The implied metal ions and coordinating groups are marked in red.

Metal Ions			Coordinating Groups		
Hard	Borderline	Soft	Hard	Borderline	Soft
Li^+^, Na^+^, K^+^, Be^2+^ Mg^2+^, Ca^2+^, Sr^2+^, Mn^2+^, Al^3+^, Ga^3+^, Cr^3+^, Fe^3+^, Sn^4+^, (CH_3_)_2_Sn^2+^, UO_2_^2+^, VO^2+^	Fe^2+^, Co^2+^, Ni^2+^, Cu^2+^, Zn^2+^, Pb^2+^, Sn^2+^, Sb^3+^, Bi^3+^	Cu^+^, Ag^+^, Au^+^, Hg^+^, Pd^2+^, Cd^2+^, Pt^2+^, Hg^2+^, CH_3_Hg^+^, Pt^4+^	H_2_O, OH^−^, F^−^, RCOO^−^, Cl^−^, RO^−^, NH_3_, RNH_2_	C_6_H_5_NH_2_	R_2_S, RSH, RS^−^

**Table 2:** Some exposure sources and target organs for Hg, Cd, and Pb.

	Important Sources of Occupational Exposure	Routes of Exposure	Important Sources of Environmental Exposure	Routes of Exposure	Target Organs of Toxicity
**Elemental mercury**	Coal-burning, waste incineration, gold extraction, dental amalgam handling, fluorescent lamp manufacturing	Inhalation	Dental amalgam in teeth	Inhalation	Central and peripheral nervous system
**Inorganic mercury salts**	-	-	Use of skin lightening products and medicinal use of mercury salts	Gastrointestinal ingestion, transdermal	Kidneys
**Methyl mercury**	-	-	Food (fish, seafood)	Gastrointestinal ingestion	Central nervous system
**Cadmium**	Production of nickel-Cd batteries, Cd plating, Cd-containing paint production	Inhalation	Food (rice, potato, and wheat, offal, seafood) Tobacco smoke	Gastrointestinal ingestion Inhalation	Kidneys Skeleton
**Lead**	Mining, smelting, battery manufacturing, traditional printing technology	Inhalation	Food, drinking water, dust and soil (in children)	Gastrointestinal ingestion	Central nervous system, hematopoietic system, kidneys

**Table 3:** Protonation constants of thioglycolic acid, thiomalic acid, meso-2,3-dimercaptosuccinic acid (DMSA), dimercaptopropane sulfonate (DMPS), penicillamine, lipoic acid, dihydrolipoic acid, and some other simple ligands useful to characterize the acid behavior of SH ligands.

Structure	Name	Acronym	Formula	MW	log K_1_	log K_2_	log K_3_	log K_4_
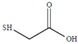	Thioglycolic acid	TGA	C_2_H_4_O_2_S	92.11	[174] 9.96	3.44		
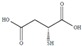	Thiomalic acid	TMA	C_4_H_6_O_4_S	150.15	[174] 10.24	4.52	3.12	
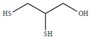	2,3 Dimercapto propan-1-ol	BAL	C_3_H_8_OS_2_	124.23	10.8	8.7		
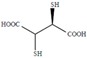	meso-Dimercapto succinic acid	DMSA	C_4_H_6_O_4_S_2_	182.22	[174] 12.05	9.65	3.43	2.71
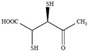	Methyl-DMSA	MDMSA	C_5_H_8_O_4_S_2_	196.22	[174] 9.98	7.15	2.8	
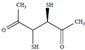	Dimethyl-DMSA	DDMSA	C_6_H_10_O_4_S_2_	210.22	[174] 7.31	5.23		
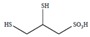	Unitiol	DMPS	C_3_H_8_O_3_S_3_	188.289	[175] 11.38	8.69		
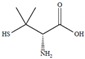	d-Penicillamine	DPEN	C_5_H_11N_O_2_S	149.212	[176] 10.35	7.91	2.19	
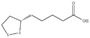	Lipoic acid	LA	C_8_H_14_O_2_S_2_	206.343	[177] 6.37 * ^$^ 4.704(1)			
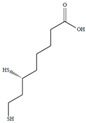	Dihydrolipoic acid	DHLA	C_8_H_16_O_2_S_2_	208.343	[178] 11.02	9.86	4.73	

The protonation constants related to the SH groups are marked in red. * Determined in non-aqueous solvent, ^$^ determined in this work.

**Table 4:** Complex formation constants of thioglycolic acid, thiomalic acid, DMSA, DMPS, penicillamine, lipoic acid, dihydrolipoic acid with Hg^2+^, Cd^2+,^ and Pb^2+^. The pM values for each system are also reported in red.

	Hg^2+^		Cd^2+^		Pb^2+^			
Ligand	Species	Logβ	Species	Logβ	Species	Logβ	Temp./Ion. Str.	Method
**TGA**	[186] HgL	34.5	[187] CdLH	11.08	[188] PbL	8.5	[186] 25, 0.1 M NaClO_4_	EMF
	HgL_2_	40.5	CdL	4.34			[187] 25, 3 M LiClO_4_	gl
			CdL_2_	6.49			[188] 25, 0.15 M	gl
**pM**	**32.9**		**6.00**		**6.95**			
**TMA**	[189] HgL	9.94	[190] CdL	10.05	[181] PbL	10.80	[189] 25, 0.1 M KNO_3_	gl
	HgL_2_	18.07	CdL_2_	13.51			[190] 25, 0.2 M KNO_3_	gl
			Cd_3_L_4_	41.59			[181] 30, 0.007 ClO_4_^−^	gl
**pM**	**8.24**		**7.78**		**8.90**			
**DMSA**	§	----	[181] CdLH_3_	28.73	[191] PbL	17.4	[181] 25, 0.1 M KCl	gl
			CdLH	23.50			[191] 25, 0.1 M	Spect.
			CdL	17.11				
**pM**			**11.48**		**11.45**			
**DMPS**	[192] HgL	42.2	[193] CdL_2_	28.27	[181] PbL	16.38	[192] 25, 0.1 M NaClO_4_	ISE
	HgL_2_	53.1	Cd_3_L_3_	59.9	PbL_2_	22.21	[193] 25, 0.2 M KNO_3_	gl
			Cd_3_L_4_	71.9			[181] 20, 0.1 KNO_3_	EMF
			Cd_5_L_6_	114.3				
			Cd_7_L_8_	156.7				
**pM**	**37.60**		**13.24**		**12.00**			
	[181] HgL	39.71	[181] CdL	17.32			[181] 20, 0.1 KNO_3_	EMF
			CdL_2_H	35.19			[181] 37, 0.15 NaCl	gl
			CdL_2_	28.22				
			Cd_2_L2	37.72				
			Cd_3_L_3_H	61.91				
**pM**	**34.80**		**12.14**					
**DPEN**	[194] HgL	37.6	[190] CdL	11.53	[181] PbLH	15.87	[194] 25, 0.1 M KNO_3_	
	HgL_2_H	52.31	CdL_2_	19.64	PbL	13.12	[190] 25, 0.2 M KNO_3_	gl
	HgL_2_	43.69	Cd_3_L_4_	50.22	PbL_2_H	26.19	[181] 25, 0.1 M KCl	gl
					PbL_2_	17.7		
**pM**	**34.50**		**8.61**		**10.50**			
	[186] HgL	37.8	[194] CdL	11.51			[186] 25, 0.1 M NaClO_4_	gl
	HgL_2_H	53.6	CdL_2_H	15.94			[194] 25, 0.1 M KNO_3_	
	HgL_2_	44.50	CdL_2_	19.52				
			CdL_3_	22.35				
**pM**	**35.30**		**8.46**					

§ Precipitation occurs [195].

**Table 5:** Recommended chelation treatment in poisonings with mercury, cadmium, and lead.

Toxic Agent	Recommended Chelation Treatment
Inorganic mercuric salts	DMPS
Methyl mercury	DMSA
Elemental mercury vapor	DMPS (initially combined with BAL)
Cadmium	DMSA (combined with MiADMSA)
Lead	DMSA (combined with Monensin)

## Supplementary Materials

Supplementary materials are available online.
