# Supplementary material for: A Review on Coordination Properties of Thiol-Containing Chelating Agents Towards Mercury, Cadmium, and Lead
Source: Molecules. 2019 Sep 6;24(18):3247. doi: 10.3390/molecules24183247 (PMC6767255; doi:10.3390/molecules24183247)
Supplement: Supplementary file 1 [file molecules-24-03247-s001.pdf]

Table 1S. Structures of the ligands. Hydrogen in light grey, carbon in grey, nitrogen in blue, sulfur in yellow and oxygen in red. Coordinates obtained from the Cambridge Structural Database, image created with Mercury3.5.

**CCDC-Refcode Reference**	**Structures**	**Ligand**	
BECVUC<br><br>K. Igawa, N. Yoshinari, T. Konno, Acta Crystallogr.,Sect.C:Cryst.Struct.Commun.<br><br>68 (2012) 6332	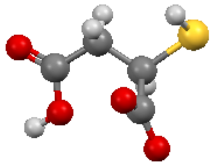	TMA	
JUBFER<br><br>G.J.Pyrka, N.Scott, Q.Fernando, Acta Crystallogr.,Sect.C:Cryst.Struct.Commun. 48 (1992) 2007	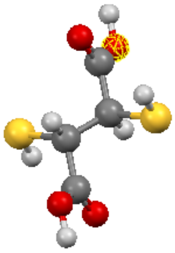	DMSA	
CEDFAS<br><br>H.E. Howard-Lock, C.J.L. Lock, P.S. Smalley, J.Crystallogr.Spectrosc.Res., 13 (1983) 333	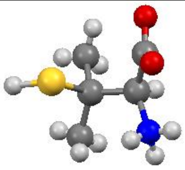	DPEN	
HAXJIB<br><br>C.-P.Racz, G.Borodi, M.M.Pop, I.Kacso, S.Santa, M.Tomoaia-Cotisel, Acta Crystallogr.,Sect.B:Struct.Sci., 68 (2012) 164	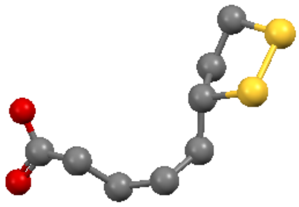	LA	

Table 2S. Structures of Hg^{2+} with different ligands bearing mercapto groups. Hydrogen in light grey, carbon in grey, nitrogen in blue, sulfur in yellow, oxygen in red and mercury in light blue. Coordinates obtained from the Cambridge Structural Database, image created with Mercury3.5.

**CCDC-Refcode Reference**	**Hg^{2+} Structures**	**Complex**	**Ligand**
AQONOJ<br><br>J.M. Bramlett, Hee-Jung Im, Xiang-Hua Yu, Tianniu Chen, Hu Cai, L.E. Roecker, C.E. Barnes, Sheng Dai, Zi-Ling Xue, Inorg. Chim. Acta, 317 (2004) 243	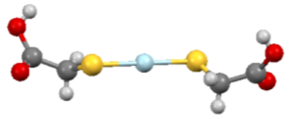	HgL_2	TGA<br><br>$COOHCH_2$-SH
BEPQAO<br><br>G.G. Hoffmann, I. Steinfatt, W. Brockner, V. Kaiser, Z. Naturforsch. B:Chem.Sci., 54 (1999) 887	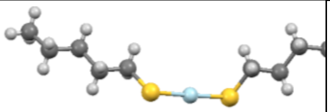	HgL_2	$CH_3(CH_2)_3CH_2SH$
CAYFUD<br><br>Z. Popovic, D. Matkovic-Calogovic, J. Hasic, M. Sikirica, D. Vikic-Topic, Croat. Chem. Acta, 72 (1999) 279	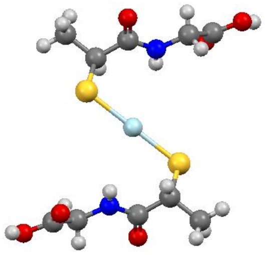	HgL_2	HCOO-CH_2-NH-CO-CH-(CH_3)-SH
HAYQEE<br><br>H.Fleischer, Y.Dienes, B.Mathiasch, V.Schmitt, D.Schollmeyer, Inorg. Chem., 44, (2005) 8087	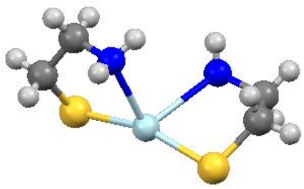	HgL_2	NH_2-CH_2-CH_2-SH
MERSET01<br>K.A. Fraser, W. Clegg, D.C. Craig, M.L. Scudder, I.G. Dance, Acta Crystallogr. ,Sect.C:Cryst. Struct. Commun., 51 (1995) 406	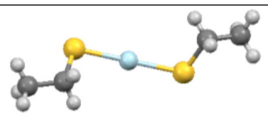	HgL_2	CH_3-CH_2-SH
AHOQOE<br><br>Kuan-Yi Wu, Chang-Chih Hsieh, Yih-Chern Horng, J.Organomet.Chem., 694 (2009) 2085	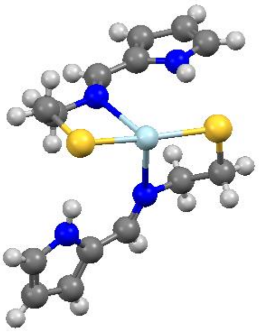	HgL_2	R-CH_2-NH-CH_3-CH_2-SH

MECBHG<br>C. Perchard, G. Zuppiroli, P. Gouzerh, Y. Jeannin, F. Robert, J. Mol. Struct., 72 (1981) 119	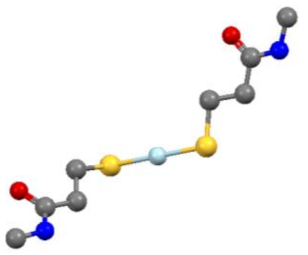	HgL_2	CH_3-NH-CO-CH_2-CH_2-SH^-
MERMES<br>D.C. Bradley, N.R. Kunchur, J. Chem. Phys., 40 (1964) 2258	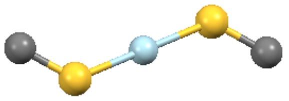	HgL_2	CH_3-SH
TEVQOA01<br>R.D.Schluter, G.Krauter, W.S.Rees Junior, J.Cluster Sci., 8 (1997) 123	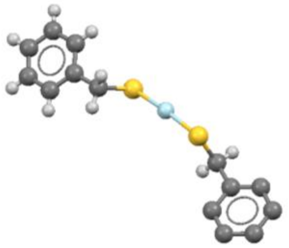	HgL_2<br>-	Φ-CH_2-SH
VOLFOR<br>I. Casals, P. Gonzalez-Duarte, W. Clegg, Inorg. Chim. Acta, 184 (1991) 167	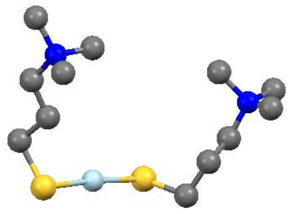	HgL_2	$N(CH_3)_3$-CH_2-CH_2-CH_2S
XIJKOQ<br>Chong-Hyeak Kim, S. Parkin, M. Bharara, D. Atwood, Polyhedron, 21 (2002) 225	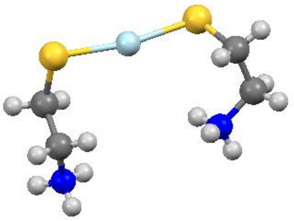	HgL_2	NH_3-CH_2-CH_2S
DAXPUN<br>G. Henkel, P. Betz, B. Krebs, Chem. Comm.,(1985) 1498	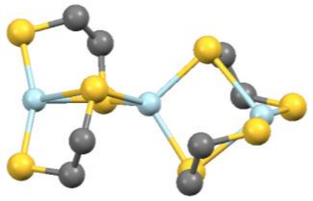	Hg_3L_4	HS-CH_2-CH_2-SH
WACXAZ<br>N. Govindaswamy, J. Moy, M. Millar, S.A. Koch, Inorg. Chem., 91 (1992) 5343	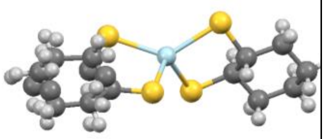	HgL_2	$C_6H_{12}S2$

Table 3S. Structures of Cd^{2+} with different ligands bearing mercapto groups. Hydrogen in light grey, carbon in grey, nitrogen in blue, sulfur in yellow, oxygen in red, cadmium in pink and chloride in green. Coordinates obtained from the Cambridge Structural Database, image created with Mercury3.5.

**CCDC-Refcode Reference**	**Cd^{2+} Structures**	**Complex**	**Ligand**
CAHGUN<br>I.G.Dance, M.L.Scudder, R.Secomb, Inorg. Chem., 22 (1983) 1794	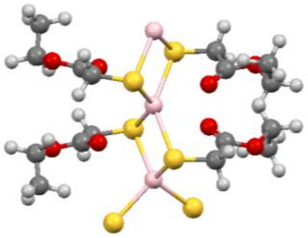	Cd_3L_6	CH_3-CH_2-O-CO-CH_2SH
DETHOZ<br>A.D. Watson, C.P. Rao, J.R. Dorfman, R.H. Holm, Inorg. Chem., 24 (1985) 2820	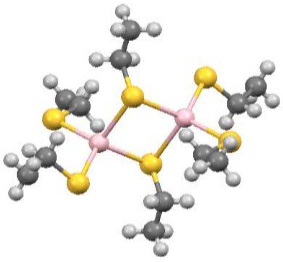	Cd_2L_6	CH_3-CH_2-SH
DUFZAF<br>C.P. Rao, J.R. Dorfman, R.H. Holm. Inorg. Chem., 25 (1986) 428	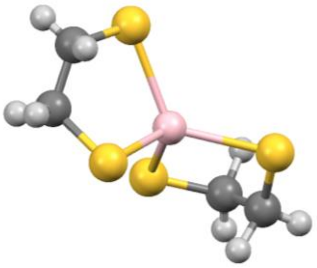	CdL_2	SH-CH_2-CH_2-SH
HAZGAR<br>H. Fleischer, Y. Dienes, B. Mathiasch, V. Schmitt, D. Schollmeyer, Inorg. Chem., 44 (2005) 8087	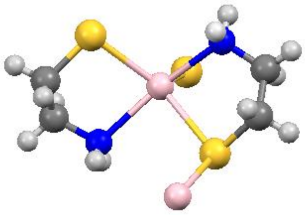	CdL_2	SH-CH_2-CH_2-NH_2
HEQMAT<br>U. Florke, CSD Communication (Private Communication), 2013	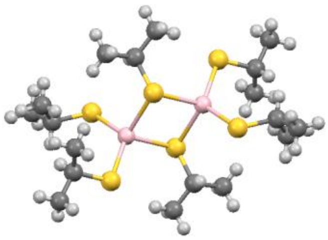	Cd_2L_6	$(CH_3)_2$-CH-SH

JIZWIY<br><br>I. Casals, P. Gonzalez-Duarte, W. Clegg, C. Foces-Foces, F.H. Cano, M. Martinez-Ripoll, M. Gomez, X. Solans, J. Chem. Soc., Dalton Trans., (1991) 2511	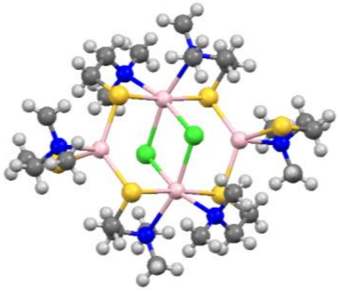	$Cd_4L_6Cl_2$	$(CH_3)_2$-N-CH-CH_2-SH
TUQREC<br><br>H. Strasdeit, A. von Dollen, A.-K. Duhme, Z. Naturforsch., B:Chem.Sci., 52 (1997) 17	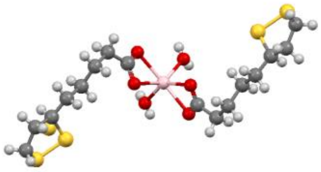	$CdL_2\ 2H_2O$	Lipoic acid
WACXED<br><br>N. Govindaswamy, J. Moy, M. Millar, S.A. Koch, Inorg. Chem., 31 (1992) 5343	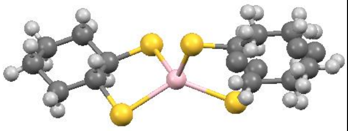	CdL_2	Cicloesene-$(SH)_2$
XICZEO<br><br>M. Mikuriya, Xiao Jian, S. Ikemi, T. Kawahashi, H. Tsutsumi, A. Nakasone, Jong-Wan Lim, Inorg. Chim. Acta, 312 (2001) 183	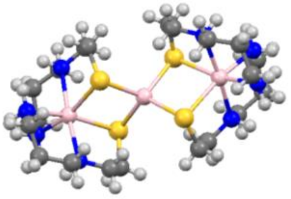	Cd_3L_4	NH_2-$CH_2CH_2CH_2$NHCH-CH_2-SH

Table 4S. Structures of Pb^{2+} with different ligands bearing mercapto groups. Hydrogen in light grey, carbon in grey, nitrogen in blue, sulfur in yellow, oxygen in red, lead in light green and chloride in green. Coordinates obtained from the Cambridge Structural Database, image created with Mercury3.5.

CCDC-Refcode<br>Reference	Pb^{2+} Structures	Complex	Ligand
DISWIL<br><br>P.A.W.Dean, J.J.Vittal, N.C.Payne, Inorg. Chem., 24 (1985) 3594	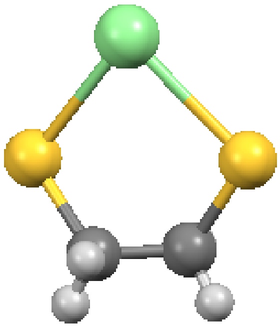	PbL	$HS\text{-}CH_2\text{-}CH_2\text{-}SH$
KEJCOS<br><br>M.S. Bharara, S. Parkin, D.A. Atwood, Inorg. Chim. Acta, 359 (2006) 3375	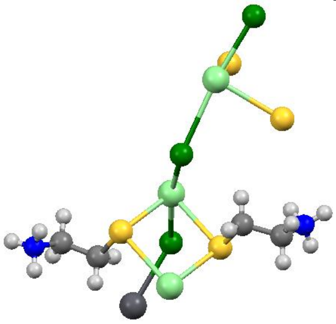	Pb_2L_2	$CH_2SH\text{-}CH_2NH_2$
DPENPB01<br><br>A.C.Schell, M.Parvez, F.Jalilehvand, Acta Crystallogr.,Sect.E:Struct.Rep.Online, 68 (2012) m489	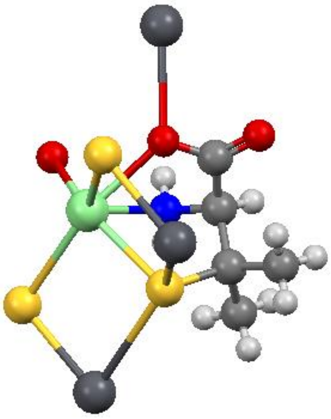	PbL	DPEN
NUFQEL01<br><br>V.N. Khrustalev, R.R. Aysin, I.V. Borisova, A.S. Peregudov, L.A. Leites, N.N. Zemlyansky, Dalton Trans., 39 (2010) 9480	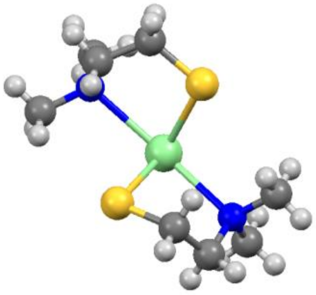	PbL_2	$HS\text{-}CH_2\text{-}CH_2N(CH_3)_2$

Fig. 1S. Speciation plots of the metal complexes whose stability constants are reported in Table 3, calculated with Hyss program at metal concentration 0.001 M and ligand concentration 0.002 M.

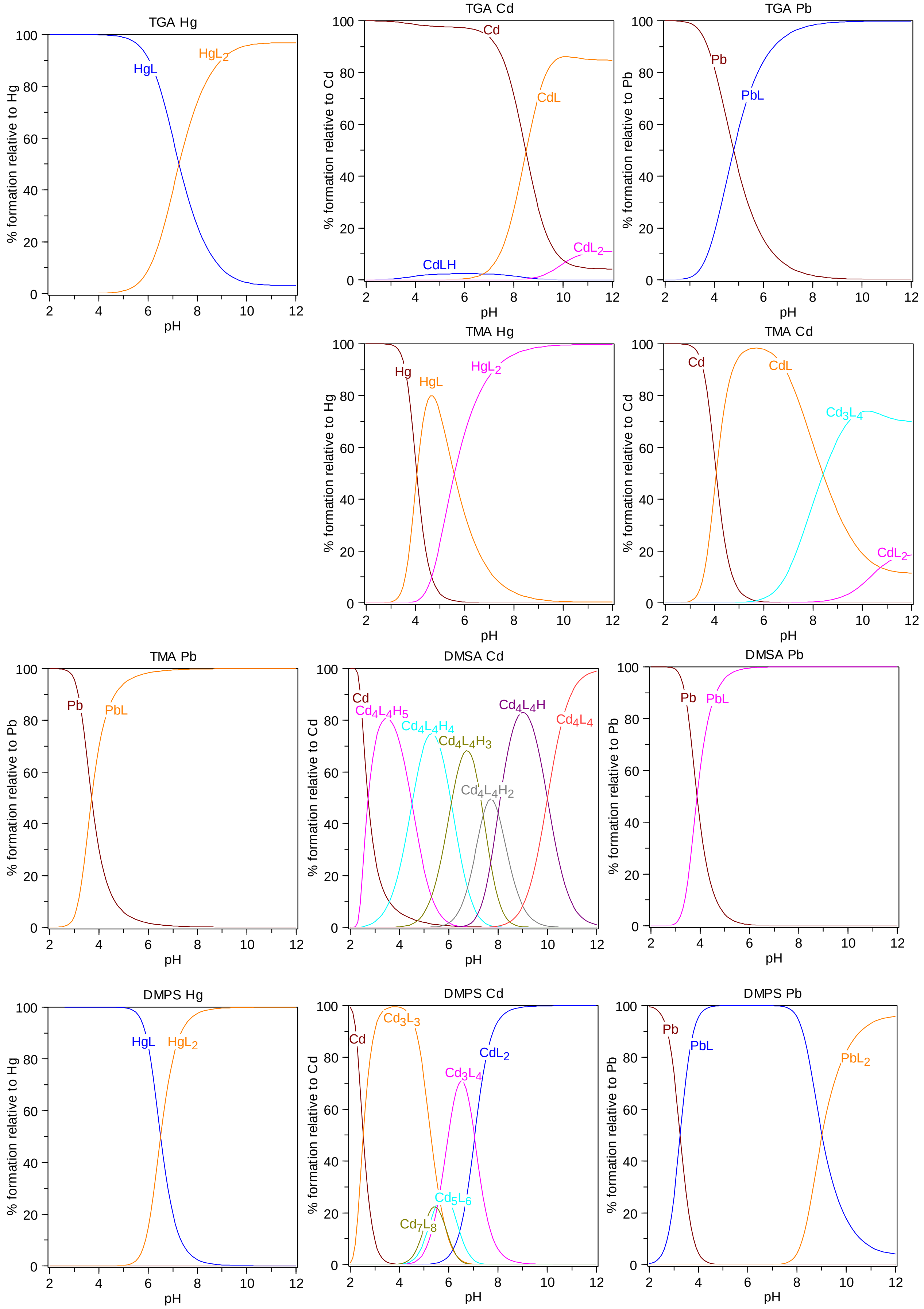

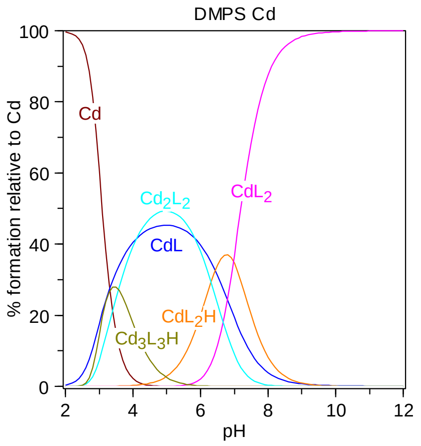
DMPS Cd
% formation relative to Cd
pH
Cd
Cd2L2
CdL2
CdL
CdL2H
Cd3L3H

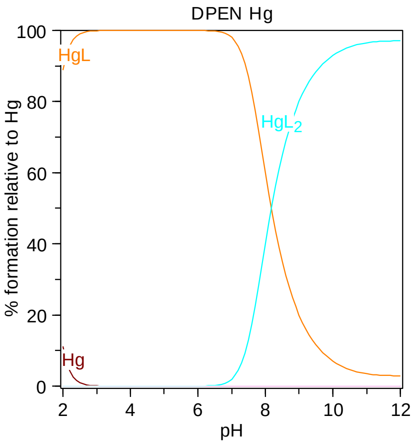
DPEN Hg
% formation relative to Hg
pH
HgL
HgL2
Hg

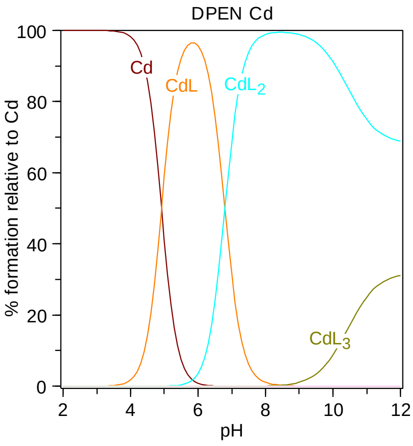
DPEN Cd
% formation relative to Cd
pH
Cd
CdL
CdL2
CdL3

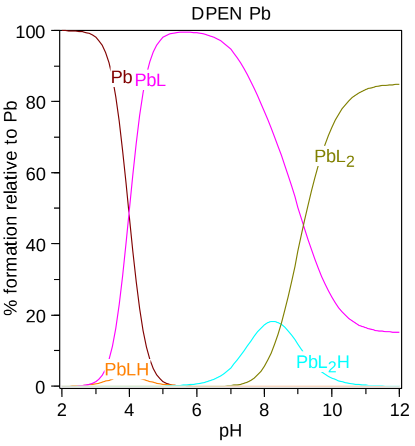
DPEN Pb
% formation relative to Pb
pH
Pb
PbL
PbL2
PbLH
PbL2H

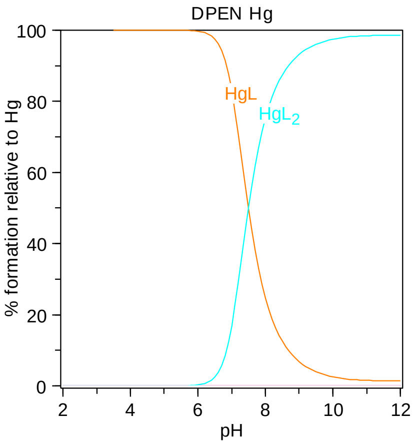
DPEN Hg
% formation relative to Hg
pH
HgL
HgL2

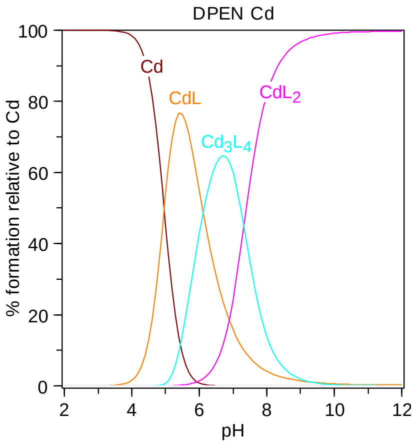
DPEN Cd
% formation relative to Cd
pH
Cd
CdL
CdL2
Cd3L4
